# A pilot study on quality of artesunate and amodiaquine tablets used in the fishing community of Tema, Ghana

**DOI:** 10.1186/1475-2875-12-220

**Published:** 2013-06-28

**Authors:** Andrews O Affum, Samuel Lowor, Shiloh D Osae, Adomako Dickson, Benjamin A Gyan, Delali Tulasi

**Affiliations:** 1 Nuclear Chemistry and Environmental Research Centre, National Nuclear Research Institute, Ghana Atomic Energy Commission, P.O. Box LG 80, Legon, Accra, Ghana; 2 Cocoa Research Institute of Ghsana, P.O. Box 8, New Tafo-Akim, Ghana; 3 Department of Immunology, Noguchi Memorial Institute for Medical Research, P.O. Box LG 581, Legon, Accra, Ghana

**Keywords:** Artesunate and amodiaquine tablets, Counterfeit/substandard/falsified, Ferric chloride test, Cobaltous thiocyanate test, Titrimetric, Spectrophotometry, High performance liquid chromatography

## Abstract

**Background:**

The ineffectiveness of artesunate and amodiaquine tablets in malaria treatment remains a health burden to WHO and governments of malaria-endemic countries, including Ghana. The proliferation of illegitimate anti-malarial drugs and its use by patients is of primary concern to international and local drug regulatory agencies because such drugs are known to contribute to the development of the malaria-resistant parasites in humans. No data exist on quality of these drugs in the fishing village communities in Ghana although the villagers are likely users of such drugs. A pilot study on the quality of anti-malarial tablets in circulation during the major fishing season at a malarious fishing village located along the coast of Tema in southern Ghana was determined.

**Methods:**

Blisterpacks of anti-malarial tablets were randomly sampled. The International Pharmacopoeia and Global Pharma Health Fund Minilab protocols were used to assess the quality of anti-malarial tablets per blisterpacks allegedly manufactured by Guilin Pharmaceutical Co Ltd, China (GPCL) and Letap Pharmaceuticals Ltd, Ghana (LPL) and sold in chemical sales outlets at Kpone-on–Sea. Ferric chloride and cobaltous thiocyanate tests confirmed the presence of active ingredients in the tablets. A confirmatory test for the active ingredient was achieved with artesunate (ICRS1409) and amodiaquine (ICRS0209) reference standards. A high performance liquid chromatography analysis confirmed the amount of artesunate found in tablets.

**Results:**

Based on the International Pharmacopoeia acceptable range of 96/98 to 102% for genuine artesunate per tablet, 10% [relative standard deviation (RSD): 3.2%] of field-selected artesunate blisterpack per tablets manufactured by GPCL, and 50% (RSD: 5.1%) of a similar package per tablet by LPL, passed the titrimetric test. However, 100% (RSD: 2.2%) of amodiaquine blisterpack per tablet by GPCL were found to be within the International Pharmacopeia acceptable range of 90 to 110% for genuine amodiaquine in tablet, whilst 17% of a similar package per tablet by LPL failed spectrophotometric testing.

**Conclusion:**

Inadequate amounts of artesunate and amodiaquine detected in the tablets suggest that both pharmaceutical companies may not be following recommended drug formulation procedures, or the active pharmaceutical ingredients might have been degraded by improper storage conditions. Thus, drugs being sold at Kpone-on-Sea, Ghana may likely be classified as substandard drugs and not suitable for malaria treatment.

## Background

Counterfeit or substandard anti-malarial drug proliferation on the Ghanaian market is a public health concern to the individual, the community and government. Malaria is endemic in sub-Saharan Africa, which includes Ghana [1]. The World Health Organization (WHO) reports that in 2010 malaria caused an estimated 655,000 deaths worldwide, with most of the cases occurring among African children [2]. The report predicts that over 250 million new cases of malaria occur each year and about half the world’s population is at risk of malaria. This public health hazard has attracted attention from WHO and country-specific drug and healthcare regulatory agencies that seek to reverse the trend of anti-malarial drug counterfeiting. WHO defines counterfeit drugs as: “*one which is deliberately and fraudulently mislabelled with respect to identity and/or source. Counterfeiting applies to both branded and generic products; counterfeit products may include products with the correct ingredients or with the wrong ingredients, without active ingredients, with insufficient active ingredient or with fake packaging*” [3,4]. Substandard drugs are genuine but may not meet drug quality specifications claimed by the manufacturer and those set by WHO. Further, substandard drugs occasionally contain sub-therapeutic amounts of active pharmaceutical ingredients (API) and/or may show suboptimal release of API (dissolution). Thus, exposing parasites to sub-lethal concentrations of API(s). Nevertheless, the percent API in genuine medicines may also be reduced if they are degraded by extremes of temperature and humidity.

The use of substandard anti-malarial drugs is known to contribute to the increasing population of drug-resistant *Plasmodium falciparum*, because inaccurate dose of the active pharmaceutical ingredient (API) in the tablets are not sufficient to effectively clear the parasite from the infected individual’s body system. In Ghana, the use of chloroquine for malaria treatment has been banned because chloroquine is not effective in clearing *P. falciparum* in an infected person. For this reason, most malaria-endemic countries have adopted artemisinin-based combination therapy [artesunate (AS)–amodiaquine, AS–mefloquine, AS–sulphadoxine/pyrimethamine)] as first-line treatment of *P. falciparum* infections [5].

The global anti-malarial drug policy for Ghana requires that uncomplicated *P. falciparum* malaria infections are treated with a combined therapy of artesunate-amodiaquine and artemether-lumefantrine [6,7]. However, in cases of failed treatment in pregnancy and severe malaria, a single dose quinine therapy has been recommended. Further, for intermittent preventive treatment a combined therapy of sulphadoxine-pyrimethamine must be used. This has made artesunate-amodiaquine tablets a target of the very sophisticated and highly profiteering substandard or counterfeit drug trade. The extremely complex labelling by pharmaceutical companies makes it more difficult for counterfeit anti-malarial tablets to be visually identified [8]. In a survey on proliferation of anti-malarial counterfeiting between 1999 and 2002, it was found that 38 to 53% of artesunate anti-malarial tablets produced by Indian and Chinese pharmaceutical companies were counterfeited [9]. Globally, it is estimated that more than 10% of drugs that are traded are counterfeits [10,11]. It is possible that most of these counterfeit anti-malarial tablets are sent to underdeveloped and malaria-endemic countries, including Ghana. Although no data have been published on the level of anti-malarial counterfeiting or substandard drugs by locally (Ghanaian) based pharmaceutical companies, their contribution to the counterfeit drug trade cannot be ignored. In a study to compare quality of drug products approved by the Stringent Regulatory Authority (SRA) and WHO, it was determined that African countries had a greater proportion of SRA or WHO approved products (31.5%) than Indian cities, but experienced higher failure rates (14.21%) than Indian cities (7.83%). Further, products made in Africa had the highest failure rate of 25.77%, followed by Chinese products at 15.74%, Indian products at 3.70%, and European/US products at 1.70%; and 17.65% for Chinese products approved by the WHO [12]. The United Nations Office on Drugs and Crime has implicated China and India as major exporters of counterfeit drugs [13-15]. Therefore, monitoring of artesunate and amodiaquine, combined or single therapy drugs, for substandard, counterfeiting and degradation is necessary to alert and prevent the spread of drug-resistant *P. falciparum*.

**Figure 1:**
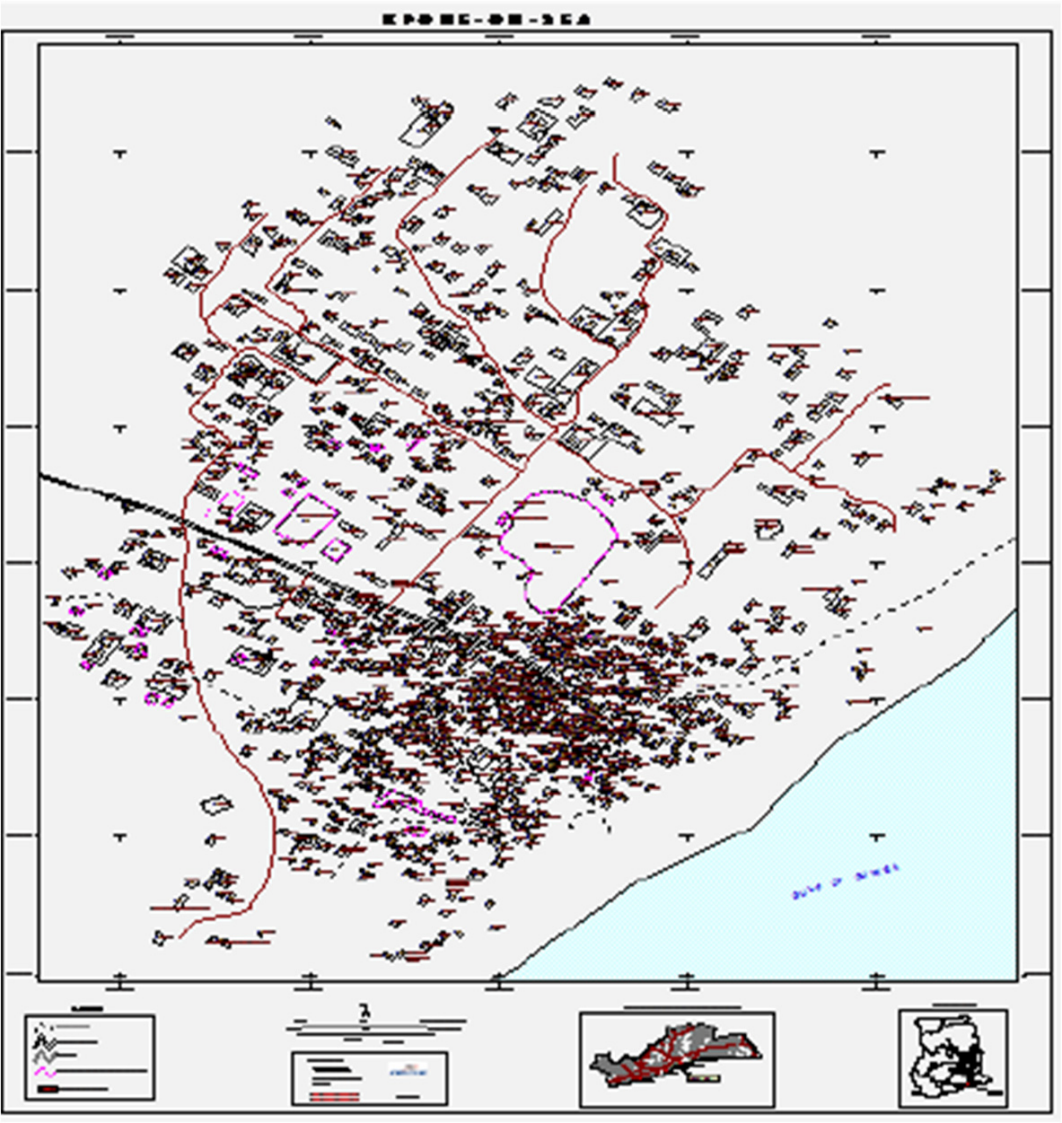
GPS map of Kpone-On-Sea showing the locations of individual houses of the study area is a map of Ghana.

In Ghana, the extent of substandard or counterfeit anti-malarial drugs has been evaluated in the major cities of Accra and Kumasi, with a reported low failure rates in all types of anti-malarials in 2010 to 54% of 2007 findings [16-18]. However, the extent of counterfeit or substandard drugs in fishing village settings in Ghana has not been assessed because information and drug regulatory enforcement is unavailable. For this reason Kpone-on-Sea (GPS map shown in Figure 1), an isolated and the largest fishing village community in Ghana, was selected as the study site. Despite the recommended methods in the International Pharmacopoeia monographs and Global Health Fund Minilab Protocols, other analytical methods such as colourimetric [19-21], liquid chromatography with ultraviolet /evaporative light scattering (LC/UV/ELS), [22-25], thin layer chromatography (TLC) [21,26-29], LC/mass spectrometry –MS [9,23,30-32], vibrational spectroscopy: FTIR/NIR, Raman and NMR [33-35] and electrochemical [36] have been used to quantitate amodiaquine and artesunate in anti-malarial tablets. However, such methods need to be validated before they can be used for drug quantitation or qualification in tablets.

The objective of this pilot study was to evaluate the quality of artesunate and amodiaquine tablets in circulation during the major fishing season at Kpone-on-Sea, a fishing village located along the coast in southern Ghana, and to assess the extent of substandard, counterfeit, or degraded anti-malarial drugs in this unique geographical location. This research is expected to encourage WHO, health organizations and governments to be effective in their drug monitoring programmes in poor communities since they are far more likely to be the victims of poor anti-malarial drugs.

## Methods

### Chemicals and reagents

All chemicals were of analytical and American Chemical Standard (ACS) grade. Hydroxylamine hydrochloride, sodium hydroxide, iron (III) chloride, phosphoric acid, hydrochloric acid, phenolphthalein and bromophenol blue, ethanol, methylene chloride, chloroform, acetone and HPLC grade methanol, toluene, acetic acid, and acetonitrile were purchased from Sigma Aldrich, St Louis, MO, USA. Artesunate reference standard (ICRS1409) and amodiaquine reference standards (ICRS0209) were purchased from the European Directorate for Quality of Medicines and Healthcare (EMDQ), Strasbourg, France. The anti-malarial drugs were purchased from all available chemical stores at Kpone-on-Sea, Tema.

### Instrumentation

UV-Vis Spectrophotometer was from Shimadzu, Japan. Microcentrifuge was from France. The high performance liquid chromatography (HPLC) system consisted of a Waters 1525 Binary Pump, Waters 2487 Dual Wavelength Absorbance Detector with an online Waters degasser and inline filter. A Waters C-18 reversed phase HPLC column of dimension 3.9 mm × 150 mm and 5 μm particle size was used for the separation. The manual injection rheodyne valve has a volume of 20 μL. The chromatograms were acquired, integrated and quantified using Waters Breeze Software version 3.3. Class-A quality glassware was used in all laboratory work.

### Study site

Kpone-on-Sea is one of the largest fishing village located in the Tema Municipal Health Directorate, within the Greater Accra Region of Ghana. It is located at 5°69’N, 0°06’E within the coastal savannah belt of West Africa and has a population of about 15,000 people. It is bordered on the east by Prampram, on the west by Tema, south by the Gulf of Guinea (Atlantic Ocean), and north by shrubland, beyond which is the Ghana Industrial Free Zone. It is at an altitude 50-100 m above sea level and has an equatorial climate. Temperatures range from 24.4 to 27.8°C with a mean of 26.1°C. Mean annual rainfall is between 1,133 and 3,606 mm with an average relative humidity index ranging from 78 to 85%. The land formation and the drainage patterns of the four sectors of the village are such that all water from the village drains into a stream that lies on the outskirts of the village. There is also a lagoon on the outskirts of the village [17]. A recent study in the village showed a prevalence of malaria (11%) with a peak parasite rate of 21% in children aged one to five years. *Plasmodium falciparum* was the major parasite detected in all positive blood slide examinations [18]. Most of the houses are constructed of cement and corrugated iron roofing. The majority of the residents (80%) are of the Ga and Ga-Adangbe ethnic groups. Most of the inhabitants are fishermen and a sizeable proportion is involved in vegetable farming (Figure 1). The rationale for selecting this study site was based on the level of education, population size, fishing activities, and reported incident rate of malaria infections amongst the population.

### Experimental design and sampling

Commercial artesunate-amodiaquine blisterpacks were purchased randomly without alerting salepersons at public chemical sales outlets of the research objective. Random drug sampling design was done by following Global Pharma Health Fund Minilab protocol, The International Pharmacopoeia and WHO recommended format for drug sampling [37]. The sampling unit for this study was the anti-malarial tablets that are sold from the various chemical outlets at Kpone-on Sea. A total of 32 blisterpacks containing both AS+AM tablets with the same lot or batch numbers manufactured by Guilin Pharmaceutical Co Ltd, China (GPCL) and Letap Pharmaceuticals Ltd, Ghana (LPL) were purchased between June and September in 2011 from 16 chemical sales outlets (two blisterpacks/chemical sale outlet). The lot or batch numbers were LQ110403A and 1370031 for GPCL and LPL, respectively. Each blisterpack by GPCL contained 12 each of artesunate and amodiaquine tablets whilst those by LPL contain six each of artesunate and amodiaquine tablets. The expiry date, manufacture date, source of drug, batch (lot) number, dose amount and the name of the chemical store sales outlet were recorded. All samples were stored at room temperature until analysis. Purchased blisterpacks manufactured by LPL were coded as “L#” whilst those by GPCL as “G#” (where # equals sample number). Each artesunate tablet manufactured by GPCL has a dose of 150 mg and those by LPL a dose of 306 mg. Similarly, each amodiaquine tablet by GPCL has a dose of 50 mg and those by LPL 100 mg. All drug samples were analysed before expiry date. Two blisterpacks of same batch number were purchased from each chemical sales outlet.

### Sample treatment

A sample was defined as one dosage unit: such as a blister or blisters in one package. Artesunate tablets were separated from amodiaquine tablets from each blisterpack and kept in a sealable rubber sachet. The total artesunate tablets per blister (equivalent to full treatment dose) were grounded in porcelain with a pestle into fine powder. Similarly, the total amodiaquine tablet per each blister was grounded into fine powder. Powdered tablets were kept in dry, sealable, rubber sachets at room temperature until analysed.

### Qualitative test

A qualitative test based on physical and visual colour formation was done by following WHO recommended protocol for artesunate and amodiaquine.

Packages were visually screened and compared to genuine samples when available to identify counterfeit drugs. Anti-malarial packages with following defects were classified as counterfeit: (1) apparent trademark violations or other obvious labelling infractions; (2) obvious poor packaging of product which suggested degraded product; (3) incorrect spelling of product name; (4) manufacturing date after the expiry date of product; (5) odd font or font size differed from innovator brands or branded generic originals; and, (6) fake holograms.

### Ferric chloride test for artesunate

This method was adapted from the methods in The International Pharmacopoeia monograph for anti-malarials [38]. Briefly, hydroxylamine hydrochloride solution was prepared from 3.5 g of hydroxylamine hydrochloride, 95 mL of 535 g/L ethanol, 0.5 mL of 1g/L bromophenol and sufficient 0.5 M potassium hydroxide/ethanol added until a greenish tint developed. The final solution was diluted to 100 mL with 535 g/L ethanol. Approximately 0.271 g tab/50 mg artesunate (AR) by GPCL and 0.178 g tab/ 50 mg artesunate (AR) by LPL was weighed into individual 50 mL falcon tubes and 20 mL of 95% ethanol was added. The mixture was shaken for 5 min, filtered and centrifuged at × 300 g for another 5 min. Approximately 10 mL of filtrate was transferred into a clean test tube. Approximately 0.25 mL of hydroxylamine hydrochloride solution was added, followed by 0.25 mL of 80 g/L sodium hydroxide. The test samples, negative and positive controls (prepared from ICRS1409) were boiled on a water bath. The test tubes were allowed to cool to ambient temperature after which two drops of 70 g/L hydrochloric acid was added. Finally, two drops of ferric chloride was added. A red-violet complex colour indicated a positive test and was confirmed from reference standard and negative control.

### Cobaltous thiocyanate test for amodiaquine hydrochloride

This method was adapted from the protocol in the International Pharmacopoeia monograph for anti-malarials [39]. Briefly, cobaltous thiocyanate reagent was prepared from 3.4 g cobalt chloride and 2.15 g of ammonium thiocyanate with double-distilled water in 50 mL volumetric flask. About 0.273 g of artesunate powder corresponding to each tablet dose was extracted with 0.1 M hydrochloric acid and centrifuged. Approximately 2 mL of supernatant was aliquoted into new tubes and a drop of cobaltous thiocyanate reagent added to form a green precipitate. The colour formed by amodiaquine reference standard (prepared from ICRS0209) and negative control was used to identify positive and negative reactions for the test samples.

### Quantitative assay

The quantitative analysis of artesunate and amodiaquine was achieved by using the individual methods in The International Pharmacopeia monographs with little modification. The methods included titrimetic, chromatography and spectrophotometry [38,40].

### Artesunate

A titrimetric method was used to determine artesunate in tablets. Briefly, an artesunate powder equivalent to 0.25 mg artesunate API was weighed into 50 mL conical flask and 25 mL of neutralized ethanol (prepared from 750 g/L ethanol, 0.5 mL phenolphthalein/ethanol and sufficient carbonate-free 0.02 mol/L sodium hydroxide to produce a faint pick colour) was added and titrated against 0.05 mol/L sodium hydroxide with two drops of 0.01g/mL phenolphthalein indicator to a permanent pick colour. Each mL of sodium hydroxide consumed was equivalent to 19.22 mg anhydrous artesunate (C_19_H_28_O_8_). Each sample was analysed in duplicate. Positive control (prepared from ICRS1409) and negative controls were titrated as the samples.

### High performance liquid chromatography (HPLC)

Artesunate in the powdered anti-malarial tablets manufactured by GPCL and LPL were determined by following a validated WHO HPLC protocol specified in the International Pharmacopoeia monograph [38]. Briefly, the HPLC analysis was done by using Waters HPLC system set-up. A Waters HPLC C-18 column of dimensions 0.39 mm × 150 mm id and 5 μm particle size was equilibrated with a mobile phase prepared from acetonitrile and phosphate buffer at pH 3 (44:56% v/v). The absorbance wavelength for detection of artesunate was set at 216 nm. Approximately 29.95 mg of pure artesunate standard, 35.6 mg of (equivalent to 10 mg of artesunate-active pharmaceutical ingredient) artesunate tablet powder by LPL and 0.27.1 mg (equivalent to 20.1 mg of artesunate) of artesunate tablet powder by GPCL were each dissolved in 10 mL of the mobile phase, centrifuged and supernatant aliquoted into clean borosilicate glass vials. Approximately 20 μL of the artesunate standard solution, filtered tablet solution were separately analysed on the HPLC instrument using an isocratic elution flow rate of 1.0 mL/min over a 10-min run time. The retention time of artesunate reference standard was used to determine the retention time of artesunate in the powdered tablets. The concentration of artesunate in each powdered tablet was calculated from a calibration graph of peak height *versus* concentration of artesunate reference standard. The reproducibility of the retention time was determined.

### Amodiaquine

A spectrophotometry method was used to determine amodiaquine content in tablets. Briefly, approximately 100 μg/mL of stock amodiaquine reference standard was prepared in 0.1 M hydrochloric acid from which a working concentration of 30 μg/mL was prepared. Approximately 0.273g/105 mg of amodiaquine powder manufactured by GPCL and 0.233g/153 mg powder by LPL were each weighed into 100 mL volumetric flask and diluted to 100 mL with 0.1 M hydrochloric acid. Each sample solution was then 100-fold diluted to a final concentration of 15 μg/mL. At a wavelength of 342 nm the optical density of the positive reference amodiaquine standard (prepared from ICRS0209) and samples was taken against a sample blank. The amount of anhydrous amodiaquine C_20_H_12_ClN_2_O in each tablet was calculated from the equation below:

$$\begin{array}{l} \mathrm{Amodiaquine}\;\mathrm{anhydrous}\left(\mathrm{mg}\right)\\ \;=\left(355.9/428.8\right)\;\left(20C\right)\left(A_{u}/A_{s}\right) \end{array}$$

Where 355.9 = molecular weight of amodiaquine anhydrous; 428.8 = molecular weight of anhydrous amodiaquine hydrochloride; C = concentration in μg/mL calculated with reference to the anhydrous substance of amodiaquine hydrochloride in the reference; A_u_ = absorbance of the solution of the substance being examined; A_s_ = absorbance of reference solution.

### Statistical analysis

Results were analysed by a one-sample t-test at confidence level of 95% and test value of 0.05, using Microsoft (Richmond, VA, USA) Statistical Package for Social Sciences (SPSS) version 16.0.

## Results

### Qualitative analysis of anti-malarial tablets manufactured by pharmaceutical companies

All the artesunate and amodiaquine tablets manufactured by GPCL and LPL were found to contain some amount of their respective API. The positive tests for the amodiaquine and artesunate tablets were confirmed with reference standards ICRS0209 and ICRS1409 respectively. The negative control test (without the active ingredients) did not form expected complex when compared to the positive test.

### Quantitative analysis of anti-malarial tablets manufactured by pharmaceutical companies

**Figure 2:**
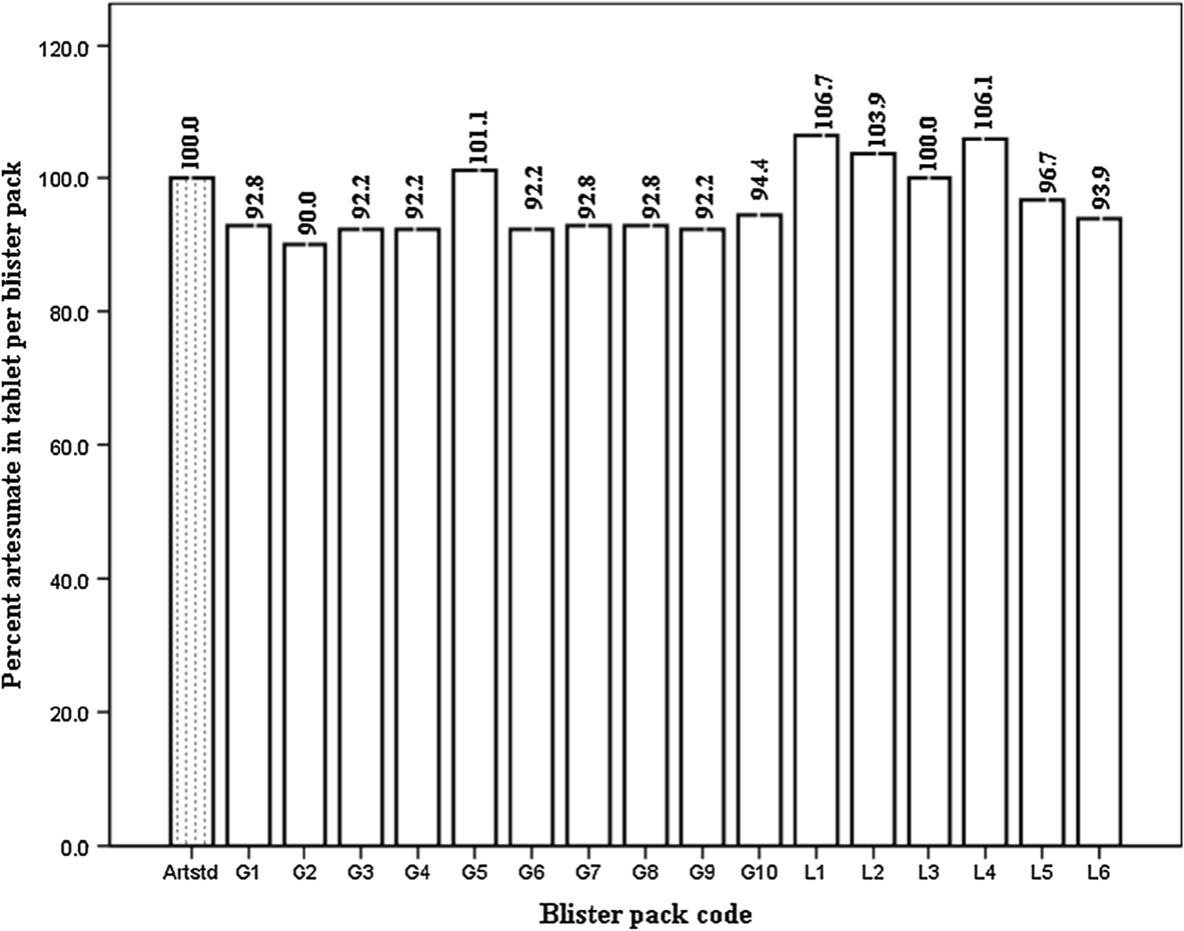
**Titrimetric analysis of artesunate API in tablets from GPCL and LPL.** Meaning of abbreviations: Artstd = artesunate reference standard; G1 to G10 = artesunate blisterpack by Guilin Pharmaceutical Co Ltd (n = 10); L1 to L6 = artesunate blister pack by Letap Pharmaceutical Ltd (n = 6); Total number of samples = 16.

Varying amount of artesunate-active ingredient in the anti-malarial tablets manufactured by both pharmaceutical companies were obtained but some APIs were within the specification range of 96 to 102% for genuine artesunate recommended by the International Pharmacopoeia (Figure 2). Artesunate content in tablet/blisterpack manufactured by GPCL varied between 90 and 101.1% of the amount of active ingredients specified on the label. Similarly, artesunate content in tablets/blisterpack manufactured by LPL varied between 93.9 and 106.7% of the label amount specified. An HPLC analysis of the tablets however confirmed the amount of artesunate in tablets obtained by the titrimetric method.

The percentage of amodiaquine-active ingredients in anti-malarial tablets manufactured by both pharmaceutical companies is shown in Figure 2. Amodiaquine content in tablets/blisterpack manufactured by GPCL varied between 99.4 and 105.4% of the amount specified on label. A wide variation of between 5.1 and 106.0 % in amount of amodiaquine-active ingredients was detected in tablets/blisterpack manufactured by LPL. The acceptable range for amodiaquine, recommended by the International Pharmacopoeia, with regard to the method used is between 90 and 110%. The results obtained from the qualitative test for both anti-malarial drugs are summarized in Table 1.

**Table 1:** Percentage of artesunate and amodiaquine anti-malarial tablet per blisterpack manufactured by GPCL and LPL determined by methods in International Pharmacopoeia monograph

**Anti-malarial blisterpack identification number**	**Percentage of artesunate per tablet (%)**		**Classification based on PhInt acceptable range of 96/98 – 102%**	**Percentage of amodiaquine per tablet (%)**	**Classification based on PhInt acceptable range of 90 – 110%**
	**HPLC**	**Titrimetric**		**Spectrophotometry**	
G1	66.4	92.8	Failed	102.3	Passed
G2	78.1	90.0	Failed	99.4	Passed
G3	85.3	92.2	Failed	104.9	Passed
G4	92.0	92.2	Failed	102.5	Passed
G5	108.9	101.1	Passed	101.5	Passed
G6	84.4	92.2	Failed	105.2	Passed
G7	109.2	92.8	Failed	105.4	Passed
G8	81.12	92.8	Failed	100.4	Passed
G9	99.3	92.2	Failed	100.5	Passed
G10	84.2	94.4	Failed	103.0	Passed
L1	102.1	106.7	Failed	103.1	Passed
L2	114.2	103.9	Failed	103.6	Passed
L3	97.1	100.0	Passed	106.0	Passed
L4	102.6	106.1	Failed	95.2	Passed
L5	93.7	96.7	Passed	5.1	Failed
L6	92.1	93.9	Failed	96.7	Passed
Artstd/Amdstd*	100	100.0	Passed	100*	Passed*

Each anti-malarial blisterpack contains amodiaquine and artesunate tablets. Meaning of abbreviations: G1 to G10 refers to anti-malarial blisterpacks obtained from GPCL, whilst L1 to L6 were obtained from LPL; Artstd refers to artesunate standard ICRS1409 obtained from EMDQ, Strasbourg, France); Amdstd* amodiaquine standard ICRS0209 obtained from EMDQ, Strasbourg, France). PhInt refers to International Pharmacopoea. Note that HPLC was not done for amodiaquine because it is not recommended in the International Pharmacopoea monograph.

## Discussion

**Figure 3:**
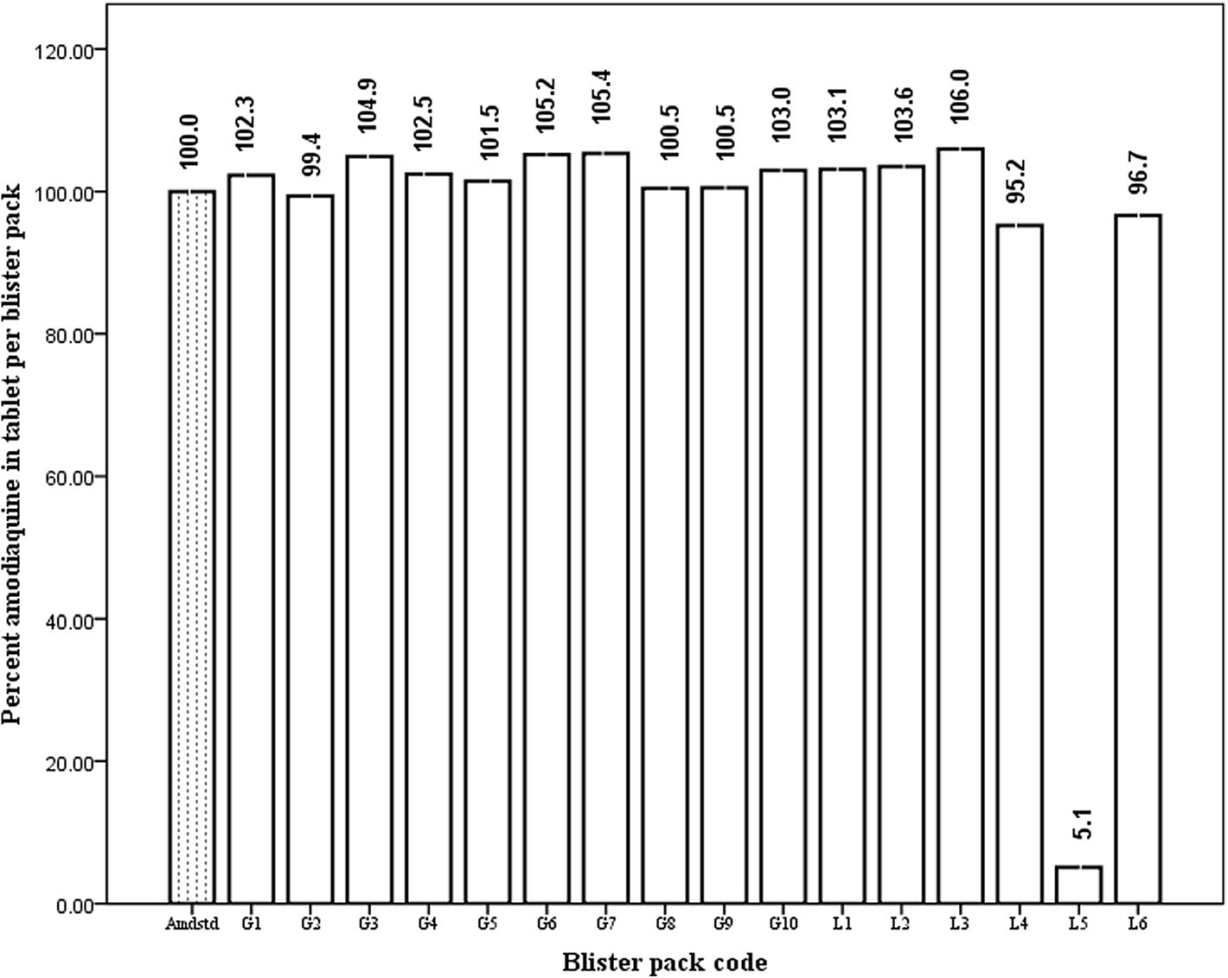
**Spectrophotometry analysis of amodiaquine API in tablets from GPCL and LPL.** Meaning of abbreviations: Amdstd = amodiaquine reference standard; G1 to G10 = amodiaquine blister pack by Guilin Pharmaceutical Co Ltd (n = 10) L1 to L6 = amodiaquine blisterpack by Letap Pharmaceutical Ltd (n = 6); Total number of samples = 16.

Physical and visual examination of the manufacturers’ print on the insert leaflet, text on package hologram and blisterpacks indicated that artesunate and amodiaquine tablets were genuinely produced by GPCL and LPL. Further, the positive confirmatory test from the qualitative analysis of both tablets manufactured by the two pharmaceutical companies confirmed their respective active ingredient in the tablets. Thus, by the above visual inspection and qualitative test, one could be led to believe that these anti-malarial tablets were genuine and had no counterfeiting characteristics. However, further analysis of the tablets found some with less active ingredient (substandard). It is important to note that anecdotal evidence from some markets supports the outcome of this work [16]. It has been determined that counterfeiters include small amounts of the active ingredient in order to pass basic qualitative test, which identifies the presence of API only and not the amount. This deception was verified in the quantitative analysis of the tablets (Table 1). From Table 2, it can be seen that anti-malarial drugs from GPCL have better precise formulation standards than those from LPL, even though both manufacturers were within the acceptable relative standard deviation (RSD) of ± 2%. It can be seen that equal amounts of excipients were added to the drugs manufactured by GPCL. The weight of excipients added may affect the release of API from the tablet when in contact with stomach fluids. Thus, API in tablets manufactured by GPCL could be more efficiently released to act on its target than API in tablets manufactured by LPL. Bio-availability and bio-equivalence problems are likely to be found in drugs formulated by LPL. Thus, tablets by GCPL were found to have uniform weight as seen in the lower RSD. Quantitative estimation of the API in both anti-malarial tablets indicated variations in the amount of the active ingredients present in each tablet per blister purchased from Kpone-on-Sea (Figures 2 and 3). Using the International Pharmacopoeia specified range of 96/98 to 102% for genuine artesunate drugs, most of the tablets were found to be substandard. About 17% of the blisterpacks manufactured by GPCL passed the International Pharmacopoeia specifications for artesunate while 83% failed, as shown in Figure 3. About 50% of artesunate tablets manufactured by LPL failed. It is important to note that substandard drug production is either unintentional or negligent and requires regulatory measures to correct [4]. The results obtained for artesunate by GPCL seem to have improved the percentage of artesunate-active pharmaceutical ingredient label claim which has been reported [25,26], as shown in Additional file 1. In a research work done in Accra, Ghana [26], only two (20%) out of 10 single-dose artesunate (active ingredients in tablets ranged between 69.3 and 92.8%), determined by the HPLC, complied with The International Pharmacopoeia standards for genuine artesunate tablets. Further, in Kumasi, Ghana [25], the artesunate content of tablets purchased from various pharmacies contained between 47.9 and 99.9% of API. The International Pharmacopoeia acceptable range for genuine amodiaquine tablet, with regard to the method used in this study, is between 90 and 110%. Using this criterion, all amodiaquine tablets by GCPL were found to be genuine whilst 17% of the tablets by LPL failed (Figures 2 and 3). The unexpectedly low amount of amodiaquine and artesunate in the tablets, judged by the standards set by The International Pharmacopeia for genuine tablets, could be due to inadequate storage conditions at high temperatures and humidity in the chemical stores, or to insufficient amount of API added during the formulation process by the manufacturers. Harsh conditions can cause drug degradation, which can lower the strength of active substance or increase degradation and toxicity. A comparison of percentage failure rate in this study to data shown in Additional file 1 indicates that percent counterfeit or substandard artesunate and amodiaquine tablets are no different from previous values obtained in sub-Saharan Africa and Southeast Asia. The RSD obtained for artesunate packs manufactured by GPCL and LPL were 5.2% and 5.1%, respectively.

**Table 2:** Weight of each anti-malaria tablet/mg dose manufactured by Pharmaceutical companies

**Name of anti-malaria tablet**	**Pharmaceutical company**	**Weight of tablet/ API dose (g/mg)**	**Number of tablet/blisterpack**	**Weight of total tablet in blisterpack per dose API (g/mg)**
Artesunate	Guilin Pharmaceutical Co., Ltd	0.271/50	12	3.27 ± 0.03/600
				(0.01, 0.8 %)†
Artesunate	Letap Pharmaceutical Company	0.356/100	6	2.13 ± 0.02/600
				(0.2, 0.9 %)†
Amodiaquine hydrochloride	Guilin Pharmaceutical Co., Ltd	0.273/150	12	3.26 ± 0.01/1800
				(0.04, 0.4 %)†
Amodiaquine hydrochloride	Letap Pharmaceutical Company	0.466/306	6	2.81± 0.01/1836
				(0.01, 0.5%)†

Meaning of abbreviations: ()†= the first value represents coefficient of variation and second value relative standard deviation.

*API* means active pharmaceutical ingredient.

The results obtained for HPLC analysis of artesunate powder confirmed the amount of active pharmaceutical ingredient obtained from the titrimetric analysis was calculated from y = 2.0E+06x. R^2^ = 1.0. A retention time of 6 min was obtained for artesunate over a total run time of 10 min. The RSD between blisterpack of anti-malarial drugs manufactured by GPCL and LPL was 2.1% and 5.1%, respectively. The high RSD for tablets manufactured by LPL could most likely be due to poor quality assurance and quality control during the drug formulation process, confirmed by the one sample student t-test analysis of the results obtained in this study. An UV/Vis spectrophotometric method was not used to determine the amount of artesunate but amodiaquine in the powdered tablet because artesunate has low molar extinction co-efficient and its UV/Vis spectra or fluorescence property is not distinct.

The amount of amodiaquine-active ingredient detected by the spectrophotometric method was not confirmed by HPLC because of the complexities often associated with the use of ion-pairs. Interestingly, the gold standard method recommended by WHO for amodiaquine is the spectrophotometric method.

The very low amodiaquine API found in amodiaquine blisterpack could be caused by poor formulation practice, poor storage conditions and packing of tablets in the blisterpack. Overall, these anti-malarial drugs with subtherapeutic amounts of active ingredients could likely lead to treatment failure, drug-resistant parasite, health hazards, spread of anti-malarial-resistant *P. falciparum,* and, finally death of the malaria-infected individuals.

## Conclusion

Quantitative analysis of tablets manufactured by GPLC, China and LPL, Ghana, which was reviewed under the International Pharmacopoeia standards for anti-malarial drugs, found variable amounts of artesunate-active ingredients in their tablets. However, amodiaquine tablets from both companies seem to indicate the expected amounts of amodiaquine-active ingredients except one blisterpack by LPL, which had exceptionally low amounts of the active ingredient. The variable amount of artesunate-active ingredient in the tablets may be due to inadequate storage conditions in chemical shops or less active ingredient added during the drug formulation process, which can confound drug quality assessment and detection of substandard drug samples. Therefore, it is recommended that the assessment on quality of artesunate and amodiaquine tablets be tested at first point-of-entry of the drugs into Ghanaian and other markets. Second, independent routine testing of these drugs be done at the dispensers, hospitals or pharmacies and results reconciled to first point-of-entry before the drugs are passed to the patient. In order to combat substandards, drug receiving points along the supply chain, such as customs border-points or at specific geographic locations where a high estimated percentage of drugs is passed, be equipped with the appropriate analytical tools to detect counterfeit or substandard drugs before they are inventoried and distributed to consumers. Although some organic impurities and degradants in amodiaquine and artesunate tablets have been found during other research work, future lines of research will look for new impurities that might be harmful to patient. The presence of unknown active ingredients was not detected in both artesunate and amodiaquine tablets although such phenomenon has been detected in some anti-malarial tablets, but before the 1999 WHO data on anti-malarial counterfeiting. Thus, this study confirms the predominant existence of substandard artesunate and amodiaquine tablets in circulation at Kpone-on-Sea in Tema. Finally, the presence of substandard artesunate and amodiaquine tablets at Kpone-on-Sea must alert health care and drug regulatory agencies to be vigilant and continually to monitor these drugs to save preventable patient mortalities. In order to prevent proliferation of poor quality artesunate and amodiaquine tablets from jeopardizing the unprecedented progress and investment made in the control and elimination of malaria infections, the police, scientists, the pharmaceutical industry, governments and WHO must work together to combat the problem. Pharmaceutical companies must be alerted about these substandard artesunate and amodiaquine so that better drug formulation procedures will be followed, as well as improvement made in the storage conditions for these drugs. Finally, the recent spread of artemisinin resistance from East Asia to African countries, present a new challenge and a danger to human health which needs to be solved.

## Competing interests

The authors declare that they have no competing interests.

## Authors’ contributions

AOA was responsible for writing the proposal, sample collection, and design of experiments in consultation with SL, AD, SDO, BAG and DT. SL, DT and AOA were responsible for the qualitative and quantitative analysis of artesunate and amodiaquine-active ingredients in anti-malarial tablets. SL and AOA are responsible for review and editing of the draft manuscript for its intellectual content. All authors contributed to interpretation of data, revision of the manuscript and gave final approval of manuscript to be published. AOA is guarantor of the paper.

## Supplementary Material

Additional file 1 **Report of poor quality artesunate and amodiaquine tablets in Southeastern Asia and sub-Saharan Africa 1999 – 2011 **[41,42,43,44,45,46,47,48,49,50,51,52,53,54]**.** Click here for file
