# Supplementary material for: A pilot study on quality of artesunate and amodiaquine tablets used in the fishing community of Tema, Ghana
Source: Malar J. 2013 Jun 28;12:220. doi: 10.1186/1475-2875-12-220 (PMC3722045; doi:10.1186/1475-2875-12-220)
Supplement: Additional file 1 — Report of poor quality artesunate and amodiaquine tablets in Southeastern Asia and sub-Saharan Africa 1999 – 2011 [41,42,43,44,45,46,47,48,49,50,51,52,53,54]. [file 1475-2875-12-220-S1.doc]

**Additional file 1 :** Report of poor quality artesunate and amodiaquine tablets in Southeastern Asia and sub-Saharan Africa 1999 – 2011

|  | Location | Date of sample collection | Drug tested | Method of testing | Obtained from | Sampling  technique | Total samples tested | Samples which failed testing | |
| --- | --- | --- | --- | --- | --- | --- | --- | --- | --- |
| Southeast Asia |  |  |  |  |  |  |  | Chemical assay | Packaging testing |
| Newton et al., 2001^41^ | Cambodia, Laos,  Myanmar, Thailand, Vietnam | 1999–  2000 | Artesunate | HPLC, colorimetric  Testing,  packaging analysis | Private pharmacies  and outlets | Convenience | 104 | 39/104  (38%) | 31/84  (38%) |
| Newton et al., 2008^42^ | Cambodia, Laos,  Myanmar, Thailand-  Myanmar border,  Vietnam | 1999–  2005 | Artesunate | HPLC, colorimetric  testing,  packaging analysis | Private pharmacies  and outlets | Convenience  And randomly | 391 | 196/391  (50%) | 195/391  (50%) |
| Dondorp et al., 2004^43^ | Cambodia, Laos,  Myanmar, Thailand,  Vietnam | 2002–03 | Artesunate | HPLC, colorimetric  testing,  packaging analysis | Public and private  pharmacies ,  outlets and  facilities | Convenience | 303 | 103/303  (34%);  99/103  (96)† | 99/303  (33%) |
| Lon et al., 2006^44^ | Cambodia | 2003 | Artesunate, | HPLC, TLC,  packaging analysis,  disintegration analysis | Public and private  pharmacies, outlets  facilities | Convenience | 81 | 16/81  (19.8) | ND |

Samples which failed quantitative test are in n/N (%). Meaning of abbreviations: HPLC=High-perfomance liquid chromatography. NS=not specified. NA=not applicable; TLC = thin layer chromatography; PA = Packaging analysis; XRD = X-ray diffraction; PA = Pollen Analysis; MS = Mass spectrometry; DT = Dissolution test

Continuation from previous page

|  | Location | Date of sample collection | Drug tested | Method of testing | Obtained from | Sampling  technique | Total samples tested | Samples which failed testing | |
| --- | --- | --- | --- | --- | --- | --- | --- | --- | --- |
| sub-Saharan Africa |  |  |  |  |  |  |  | Chemical  assay | Packaging  testing |
| US  Pharmacopeia 2004^45^ | China | 2004 | Artesunate, | HPLC, T LC, visual inspection, dissolution  analysis | NS | Convenience | 39 | 2/39  (5%) | Not  tested |
| Sengaloundeth  et al., 2009^46^ | Laos | 2003 | Artesunate | HPLC, Colorimetric  testing, MS,PA, XRD, packaging  analysis | Private pharmacies  and outlets | Stratified  random  sampling | 30 | 27/30  (90%) | 26/30  (87%) |
| Amin et al., 2005^47^ | Kenya | 2002 | Amodiaquine | HPLC, dissolution test | Private and public outlet | Convenience | 29 | 10/29  (34.5%) | Not  tested |
| Ofori-Kwakye  et al., 2008^25^ | Ghana | NP | Artesunate | HPLC ,DT, EU pharmacopeia standards | Private & public  pharmacies & outlets | Stratifi ed  random  sampling | 12 | 10/12  (83%) | Not  tested |
| Newton et al., 2011^48^ | Cameroon, DR Congo | 2007-  2008 | Artesunate, | HPLC, MS,  PA,XRD, packaging  analysis | Pharmaceutical  companies, private  and public  pharmacies | Convenience | 31 | 11/31  (35%) | 100% |

**Continuation from previous page**

|  | Location | Date of sample collection | | Drug tested | Method of testing | Obtained from | Sampling  technique | Total Samples tested | Samples which failed testing | |
| --- | --- | --- | --- | --- | --- | --- | --- | --- | --- | --- |
| sub-Saharan Africa |  | |  |  |  |  |  |  | Chemical  assay | Packaging  testing |
| Thoithi et al., 2008^49^ | Kenya | | 2001  –05 | amodiaquine | HPLC with European  pharmacopeia standards | Public and  private pharmacies | Convenience | 41 | 11/41  (27%) | Not  tested |
| Atemnkeng  et al ., 2007^50^ | Kenya,  Democratic  Republic of Congo | | 2004 | artesunate, | HPLC with European  pharmacopeia standards | Public &  private pharmacies | Convenience | 24 | 9/24  (38%) | Not  tested |
| Tipke et al., 2008^51^ | Burkina Faso | | 2006 | Artesunate,  amodiaquine | PA, DA,  colorimetric tests, TLC, UV-Vis  spectroscopy | Private and public  pharmacies or  outlets | Convenience | 9 | 0/9  (0%);  0/6  (0%) | 1/9  (11.1%)  0/6  (0%) |
| Minzi et al  2003^52^ | Tanzania | | Not  provided | amodiaquine | HPLC, dissolution tests | Public and private  outlets | Convenience | 15 | 0/15  (0%) | Not  tested |
| Kaur et al., 2008^53^ | Tanzania | | NP | amodiaquine | HPLC and dissolution  test with US  pharmacopeia standards | Private and public  pharmacies and  outlets | Random | 100 | 6/100  (7.5 %) | Not  tested |
| Bate et al., 2008^54^ | Ghana, Kenya,  Nigeria, Rwanda,  Tanzania, Uganda | | NP | Artesunate  amodiaquine | TLC or  dissolution analysis | Private pharmacies  and outlets | Convenience | 49  29 | 15/49  (31%)  14/29  (48) | Not  tested |
